# Book Reviews and Notices

**Published:** 1888-09

**Authors:** 


### BOOK REVIEWS AND NOTICES.

A PRACTICAL TEXT-BOOK OF THE DISEASES OF WOMEN. By ARTHUR H. V. LEWERS, M.D., Lond., M. R. C. P., London; Assistant Obstetric Physician to the London Hospital; Examiner in Midwifery and Diseases of Women to the Society of Apothecaries of London, etc. With illustrations. Philadelphia: P. Blakiston, Son & Co. 1888. Chicago: W. T. Keener.

Lewers' text-book possesses the merit of simplicity and brevity to an extent that casts scientific thoroughness and completeness quite in the shade. The chapter on "Rupture of the Perineum" is a marvel of condensation. The whole subject, exclusive of treatment, is included within the limits of two pages, two-thirds of which space is devoted to central rupture. The only operations hinted at are the plain triangular ones; the only suture materials mentioned are silk, fishing-gut and catgut, and the only indications given for operating are incontinence of fæces, or flatus, and the disability "on account of prolapse of the vagina, or uterus, for the patient to wear a vaginal pessary such as the ordinary India rubber ring." The chapter on "Diseases of the Fallopian Tubes" is very interesting and instructive, yet the following quotation is all that is given of the symptoms: "The patient complains of pains across the lower part of the abdomen, and it may, or may not, be worse on one side. She has dysmenorrhœa, and the regularity of the catamenia is disturbed. If the dysmenorrhœa dates from a particular confinement, or abortion, or if a history of gonorrhœa can be obtained, the probability of the tubes being diseased is strengthened." Recurrent attacks of pelvic peritonitis upon the slightest provocation; frequent alterations of patient's condition from one of comfort to misery; occasional sudden vaginal discharges followed often by relief; dragging along or reappearance of menses after their expected or temporary cessation, and attacks of rectal tenesmus or irritation might have received some notice as occasionally or frequently depending upon disease of the tubes.

Rapid dilatation by Hegar's dilators, is rightly given preference over more gradual methods, yet the existence of bladed dilators is not thought worthy of notice. Vulliet's method of dilatation by progressive plugging is not mentioned.

Version is described as a rotation of the whole uterus "round an imaginary transverse axis." Where this axis is, whether it is stationary or movable, and, if movable, whether its position changes the form of version, is left for the student to conjecture. Lateral versions and flexions are consistently ignored. As to pessaries we read, "For all ordinary cases where the use of a vaginal pessary is indicated, either the ring pessary made of watch spring covered with India rubber, or Hodge's pessary, will be found to meet every requirement." We do not object to the recommendation of these typical forms of pessaries, but rather to the dogmatic statement that they will "in all ordinary cases meet every requirement." In fact, the chief fault of the book seems to consist less in the inadequacy of the teaching than in the representation of the imperfect teaching as meeting every requirement.

Alexander's operation is rejected as dangerous and valueless, and is not described; nor is hysterectomy (either abdominal or vaginal), while the supravaginal amputation, which is recommended for all cases of carcinoma of the cervix, is given a full and satisfactory description.

In short, the book is neither a complete, even though brief, scientific exposition of gynæcology, nor is it a book of the primary class, in which the more simple and fundamental facts and truths of gynæcology are fully and adequately described for the student. It is neither a guide for the student nor practitioner, but is rather adapted to the use of the oculist, laryngologist, or other specialist who desires to have a superficial notion of gynæcology.

Many good things are in the book which testify to the originality and ability of the author, but it is unfortunate that he has endeavored in his book to make the study of gynæcology too easy, and has taken so little space for the undertaking.

ATLAS OF VENEREAL AND SKIN DISEASES; Comprising Original Illustrations and Selections from the Plates of Professor M. KAPOSI, of Vienna, Dr. J. HUTCHINSON, of London, etc., with Original text by PRINCE A. MORROW, A. M., M. D., etc. New York: Wm. Wood & Co. 1888. Fasciculi Nos. 3, 4, 5, and 6.

The new numbers of this valuable atlas, to which attention has already been called in these pages, are fully equal in point of merit to those which have preceded. The text is written with special care, and fully explains the several features of the diseases portrayed. The illustrations, both new and old, are of the highest value. And it should be said of the old that many of them appeared first in editions which are no longer accessible to the mass of American students and practitioners.

Fig. IV of plate XXVII is a fine example of the best work here shown. It illustrates an extensive cicatricial patch on the elbow of the patient, whose tuberculo-ulcerative syphiloderm is equally well displayed in the third figure on the same sheet. In the former the group of characteristic tubercles, the white, glistening, and corded cicatrix, even yet partly covered with the crusts furnished by the ulcerative process resulting in the scar, the anatomical curves of the elbow, and the flesh-tints of the arm are delicately and admirably reproduced. As we examine it, with the satisfaction which is always awakened by the study of faithful reproductions of nature, the suggestion is made that one day both scientist and artist will combine to give us a portrait of the skin where the several diseases capable of destroying its integrity have had their sway. An atlas giving us the several pictures, not of the scars of one or a few diseases, but of those of all diseases capable of leaving scars in the skin, would be a fitting complement of those now accessible.

The present work is to be commended from every point of view, and the publishers are certainly to be congratulated on the artistic merit of the plates.

FOURTEENTH ANNUAL REPORT OF THE SECRETARY OF THE STATE BOARD OF HEALTH OF THE STATE OF MICHIGAN FOR THE YEAR ENDING SEPTEMBER 30, 1886. Pp. L and 341.

Lansing, Mich.: Thorp & Godfrey, State Printers. 1888.

The excellent work which is being done in Michigan in the way of recording and reporting meteorological conditions, and contrasting them with the prevalence of various diseases, becomes increasingly impressive and valuable with the appearance of each new volume of these reports.

As is customary, the first part of this volume consists of a record of the official acts and meetings of the State Board of Health, together with reports of investigations of matters throughout the State which come under its jurisdiction.

The part of the volume which possesses the most interest for the profession at large is the contribution of the Secretary of the Board upon the causation of pneumonia. This is upon pages 246 to 324, inclusive. The number of tables and diagrams which accompany the article is very large, and the article itself is a very valuable and important contribution to the subject which it discusses.

THE APPLIED ANATOMY OF THE NERVOUS SYSTEM. A Study of this Portion of the Human Body from a Standpoint of its General Interest and Practical Utility in Diagnosis, Designed for use as a Text-Book and Work of Reference. By AMBROSE L. RANNEY, A. M., M. D. Second edition. Re-written, enlarged, and profusely illustrated. Pp. xxxv and 791. New York: D. Appleton & Company. 1888. Chicago: W. T. Keener.

He would be indeed a carping critic who failed to find warm words of praise for this book. The author was already widely and favorably known, and this his latest publication will bring him increased honor. It is well printed on good paper, and the illustrations are very numerous and excellent. Of course most of them are reproductions of the work of other men, but those which are original with the author are a great addition to the value of the book.

The general arrangement of the subject-matter is very satisfactory, and the treatment of the special topics apparently considers all of the available knowledge. The author's style is clear and attractive, and his explanations are exceptionally easy to understand. E. W.

THE YEAR-BOOK OF TREATMENT FOR 1887. A Critical Review for Practitioners of Medicine and Surgery. By many Contributors. Philadelphia: Lea Brothers & Co. 1888. Chicago: A. C. McClurg & Co.

This little manual is a valuable compilation from the domestic and foreign medical journals of the more important advances made in the treatment of disease, with a review by some most excellent authorities.

Each department of medicine and surgery has been fully and concisely treated.

Full reference has been given to every article quoted, which makes it of e pecial value to those who do not have access to the files of many medical journals. F. B.

A GUIDE TO THE PRACTICAL EXAMINATION OF URINE. For the use of physicians and students. By JAMES TYSON, M. D., Professor of General Pathology and Morbid Anatomy in the University of Pennsylvania; one of the Physicians to the Philadelphia Hospital; Fellow of the College of Physicians of Philadelphia, etc. Sixth edition. Revised and corrected. With a colored plate and wood engravings. 8vo. Pp. 253. Philadelphia: P. Blakiston, Son & Co. 1888.

That this little manual has reached the sixth edition is abundant evidence of its usefulness. It now appears again fully revised, embracing what is new and valuable pertaining to the subject, and omitting from its pages whatever has proved to be useless. The general plan and merit of the book are so well known to the profession that to outline it would be a formal introduction of an old friend.

